# Olive Cake Meal and *Bacillus licheniformis* Impacted the Growth Performance, Muscle Fatty Acid Content, and Health Status of Broiler Chickens

**DOI:** 10.3390/ani10040695

**Published:** 2020-04-16

**Authors:** Ahmed A. Saleh, Bilal Ahamad Paray, Mahmoud A.O. Dawood

**Affiliations:** 1 Department of Poultry Production, Faculty of Agriculture, Kafrelsheikh University, Kafrelsheikh 33516, Egypt; a_saleh2006@yahoo.com; 2 Department of Zoology, College of Science, King Saud University, PO Box 2455, Riyadh 11451, Saudi Arabia; bparay@ksu.edu.sa; 3 Department of Animal Production, Faculty of Agriculture, Kafrelsheikh University, Kafrelsheikh 33516, Egypt

**Keywords:** broilers, alternative ingredients, probiotics, growth, lipid peroxidation

## Abstract

**Simple Summary:**

The extraction of oils from olives usually results in large quantities of olive cake meal (OCM), which has a high nutritional value. The OCM is used successfully in livestock and poultry feeding, but due to the high fiber content, alternative methods of treating OCM must be considered. To increase the efficiency of OCM in broiler chickens’ diet, it can be mixed with suitable microorganisms with beneficial effects. Hence, the current study investigated the influence of OCM and *Bacillus licheniformis* (BL) on the growth, nutrient utilization, blood chemistry, and muscle fatty acid profile of broilers. Birds were divided into six experimental groups (control, OCM (2%), OCM (4%), BL, OCM (2%)/BL, and OCM (4%)/BL groups). The results revealed that the inclusion of BL with OCM diets improved the fat utilization and, accordingly, increased the growth, nutrient utilization, and antioxidative response in broilers.

**Abstract:**

Olive cake meal (OCM) is characterized by its high nutritional value and is used as an alternative source of protein and fats in poultry diets. However, due to the high percentage of fiber in OCM, beneficial bacteria cells are used to improve the digestion rates. Therefore, the influence of OCM and *Bacillus licheniformis* (BL) on the growth, nutrient utilization, blood chemistry, and muscle fatty acid profile of broilers was exclusively examined in this study. Three hundred and sixty birds were randomly divided into six experimental groups (6 replicates/10 birds each): Control, OCM (2%), OCM (4%), BL, OCM (2%)/BL, and OCM (4%)/BL groups. Although feed intake was not meaningfully influenced by dietary treatments, weight gain was enhanced and feed conversion ratio was reduced (*p* < 0.05). The abdominal fat was lowered in broilers fed OCM (2%), OCM (4%), OCM (2%)/BL, and OCM (4%)/BL diets without a difference to those fed BL only (*p* < 0.05). Interestingly, blood total protein, albumin, Newcastle disease (ND) titer, and high-density lipoprotein (HDL) cholesterol were significantly increased, while total cholesterol was decreased by the mixture of OCM and BL (*p* < 0.05). Muscle oleic and linoleic acids, as well as vitamin E, increased significantly in broilers fed both OCM (4%) and BL, while linolenic acid increased in all groups except those fed BL and control diets (*p* < 0.05). Liver malondialdehyde (MDA) was decreased by feeding BL or both OCM at 2% or 4% and BL (*p* < 0.05). In conclusion, the inclusion of BL to OCM diets resulted in improved fat utilization and, accordingly, enhanced growth, nutrient utilization, and antioxidative response in broilers. Based on the obtained results, it is recommended to use BL to improve the nutritional value of OCM and to increase the feed utilization of OCM by broilers.

## 1. Introduction

As a result of increasing demand, limited supply, and a dramatic increase in the prices of feed ingredients, suitable alternative sources for poultry feed have recently been intensively studied [1,2]. Feed cost may account for more than 70% of the total production costs of broilers [3,4]. Any reduction in feed costs, which still preserves the health status of broilers, is bound to have a direct positive effect on the profitability of poultry production. A considerable effort has been applied to find alternative and sustainable protein sources to be included in broilers diets [5]. In this context, among the available plant protein alternatives, olive cake meal which has high nutritional value (lipids, 13–15%, and proteins, 9–10%), with a high level of non-starch polysaccharides (NSP) (xyloglucan and xylan-xyloglucan complexes) [3,6,7,8]. The extraction of oils from olives usually results in large quantities of olive cake meal. The olive cake meal is available in several countries around the world at reasonable prices and can be used as a plant ingredient in the feed of broilers [7]. Potential problems in olive cake meal feeding exist due to the existence of fiber and high levels of unsaturated fatty acids, which can cause high fatty acid pre-oxidation, malnutrition, and lower palatability [3]. Olive cake meal was used successfully in poultry diets of up to 10% of the total ration [3,9]. To increase the efficiency of olive cake meal in broilers’ diet, it can be mixed with suitable microorganisms to obtain beneficial effects. 

A probiotic is defined as “live strains of strictly selected microorganisms which, when administered in adequate amounts, confer a health benefit on the host” [10]. Using probiotic-enriched diets is an inexpensive practice that can be adopted by both small- and large-scale farmers, and which can offer several benefits from increasing broilers’ growth to increasing immune parameters and disease resistance [11,12]. The use of probiotics has increased due to its remarkable beneficial effects on microbiota and gut health in swine [13], poultry [14], and rabbits [15,16,17]. In parallel, the significant role of probiotic bacterium on growth, intestinal microbiota, and immunological responses in broilers has been demonstrated [14,18,19]. Indeed, lactic acid bacterial species are unique strains of probiotics authorized by the Food and Drug Administration for administration in animals [10,20]. Furthermore, dietary *Bacillus licheniformis* has been shown to increase growth performance and feed efficiency due to the secretion of digestive enzymes that can increase the digestibility of nutrients in the animal’s gut [21,22,23,24,25].

This offers a new topic for researchers, where a combination of the plant by-products and the probiotics mixture is used in poultry feeds. Al-Harthi [3] concluded that there was no adverse effect on the performance of broilers when fed a dietary olive cake meal and *Saccharomyces cerevisiae* blend. Sateri et al. [9] were also able to include up to 8% olive cake meal with a digestive enzyme mixture in the diet of broilers. To date, no data are available about the use of olive cake meal mixed with *B. licheniformis* in the diet of broilers.

With the continued increase in broiler production, it is necessary to find non-traditional alternative ingredients for use in the preparation of feed [2]. Therefore, the objective of the current study was to evaluate the effects of olive cake meal mixed with *B. licheniformis* on the performance parameters, the muscle fatty acid content, and the blood parameters of broilers.

## 2. Materials and Methods

### 2.1. Birds and Experimental Design

All of the experimental procedures in this study followed the guidelines set by the Institutional Animal Care and Use Committee at Kafrelsheikh University (Number 4/2016 EC). A total of 360 one-day-old male broiler chickens (45.7 g) were placed inside a room equipped with 36 floor bins (10 birds each) (6 treatments/6 replicates each, stocking density was 10 birds/m^2^) with a chain feeder system and automatic nipple cup drinker. The bins were arranged by placing the first replicate of each treatment then the second replicate of each treatment until the sixth replicate of each treatment to follow the completely randomized design (CRD). The first group served as control and was fed basal diets without any additives. The second and third groups were fed diets containing 2% and 4% olive cake meal (OCM; Al-Sabeel Al-Gadidah Company, Tanta, Al-Gharbia, Egypt); the fourth group was fed control diets with probiotic (*Bacillus licheniformis* (BL), Dutch State Mines Company, DSM 17236; the recommended inclusion level was 100 g/ton of feed to achieve a target inclusion of 8 × 10^12^ colony-forming units (CFU)/kg); the fifth and sixth groups were fed diets containing 2% and 4% OCM with BL. The compositions of the experimental diets are presented in Table 1. *B. licheniformis* is a commercial probiotic product called GalliPro^®^ Tech DSM 17236, and this probiotic strain was isolated from soil and is a non-GMO; the recommended dose, as provided by Interpharma^®^ Company, Egypt, was used. The diets were presented to the birds ad libitum. The photoperiod was maintained as a 21 h light/3 h dark cycle. After the brooding period, the room temperature was kept between 24 and 26 °C, with relative humidity from 50% to 60% throughout the experiment.

### 2.2. Growth Performance and Carcass Parts

Bird body weight was measured individually every week. However, feed intake was measured daily (on a group basis per pen) throughout the experimental period. The feed conversion ratio per bird was calculated. At 35 days, all birds were weighed individually and sorted from the smallest to the heaviest in weight. Then, 36 birds (1 bird per replicate; 6 birds per treatment) were slaughtered and then dissected to measure the weights of the breast muscle, the thigh muscle, the liver, and the abdominal fat.

### 2.3. Blood Samples and Plasma Biochemical Analysis

At 35 days, blood samples from 36 birds (1 bird per replicate; 6 birds per treatment) were collected from the wing vein immediately before slaughtering, gathered into heparinized test tubes, and then rapidly centrifuged (3000 rpm for 20 min at 5 °C) to separate the plasma. Plasma was stored at −20 °C pending analysis. Plasma total cholesterol, high-density lipoprotein (HDL), glutamic oxalacetic transaminase (GOT), total protein, albumin and globulin, and uric acid were measured calorimetrically by using a commercial chickens’ kit (Diamond Diagnostics, Cairo, Egypt), according to the procedure outlined by the manufacturer, using spectrophotometric analysis. Serum antibody titers against Newcastle disease (ND) were determined using the hemagglutination inhibition (HI) test using standard methods qualified in the manual of the World Organisation for Animal Health (OIE) [26].

### 2.4. Muscle Biochemical Analysis

The analysis of muscle fatty acids was conducted in 36 birds (1 bird per replicate; 6 birds per treatment) from the breast muscle (pectoral superficial muscle) by gas-liquid chromatography (GLC) according to the procedure of Saleh [27]. The concentration of muscle vitamin E and liver malondialdehyde (MDA) was determined according to Ohkawa et al. [28].

### 2.5. Statistical Analysis

The differences between the treatment groups and the control group were analyzed with a General Linear model using SPSS (version 17.0: SPSS Inc., Chicago, USA). Two-way ANOVA was applied to determine the effects of *B. licheniformis* supplementation (BL), olive cake meal inclusion (OCM), and their interaction (BL × OCM) (2 × 3 factorial design). Duncan’s new multiple range tests were used to identify which treatment conditions were significantly different from each other at a significance level of *p* < 0.05.

## 3. Results

### 3.1. Growth Performance and Organ Weight

Body weight gain (WG), feed conversion ratio (FCR), and abdominal fat were significantly influenced by OCM, BL, and their interaction (*p* < 0.05) (Table 2). Body WG showed higher (*p* < 0.05) levels in broilers fed OCM (4%), OCM (2%)/BL, and OCM (4%)/BL than those fed the control, while no difference (*p* > 0.05) was observed between the other groups (Table 2). Broilers fed OCM at 2% or 4% with BL showed a reduced FCR (Table 2). The highest WG and the lowest FCR were observed in birds fed both OCM (4%) and BL (Table 2).

The abdominal fat was decreased (*p* < 0.05) in broilers fed OCM (2%), OCM (4%), OCM (2%)/BL, and OCM (4%)/BL diets without a difference to those fed OCM (2%) (Table 2).

### 3.2. Biochemical Parameters

Blood total protein, albumin, total cholesterol, HDL cholesterol, and ND titer were significantly influenced by OCM, BL, and their interaction (*p* < 0.05) (Table 3). Blood total protein increased by feeding both BL and OCM at 2% or 4% when compared with those fed the control or BL without a difference to OCM at 2% or 4% (*p* > 0.05) (Table 3). The albumin content increased in the BL and OCM (4%) groups with regard to the control (*p* < 0.05). Interestingly, the ND titer was influenced by OCM, BL, and their mixture (*p* < 0.05). Blood total cholesterol decreased in those fed OCM (4%), OCM (2%)/BL, and OCM (4%)/BL, while HDL cholesterol increased in those fed OCM (2%), OCM (4%), OCM (2%)/BL, and OCM (4%)/BL (*p* < 0.05) (Table 3). However, GOT and uric acid were not affected by the test diets (*p* > 0.05) (Table 3).

### 3.3. Muscle Fatty Acid Profiles

**Figure 1:**
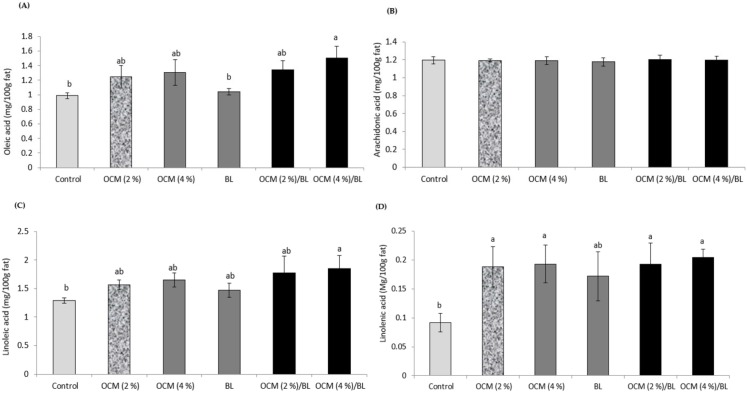
Effects of feeding *Bacillus licheniformis* (BL) or/and olive cake meal (OCM) on muscle fatty acids (oleic acid, arachidonic acid, linoleic acid, and linolenic acid) in broilers.

Muscle oleic, linoleic, and linolenic acids were influenced by OCM, BL, and their interaction (*p* < 0.05) (Figure 1; Table 4). Muscle oleic and linoleic acids increased significantly in broilers fed both OCM (4%) and BL, while linolenic acid increased in all groups except those fed BL and control diets (*p* < 0.05) (Figure 1). However, arachidonic acid was not affected by the test diets (*p* > 0.05).

### 3.4. Muscle Vitamin E and Liver MDA

**Figure 2:**
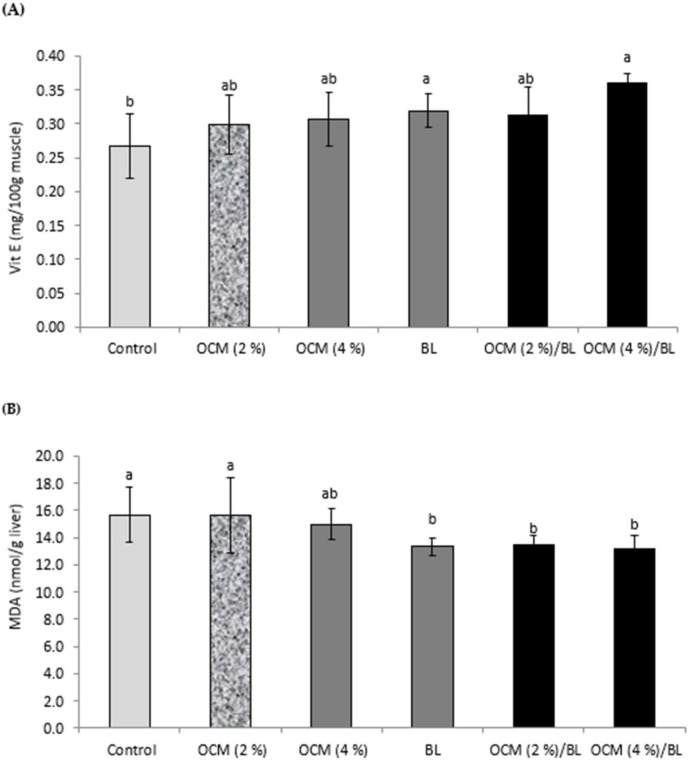
Effects of feeding *Bacillus licheniformis* (BL) or/and olive cake meal (OCM) on muscle vitamin E and liver malondialdehyde (MDA) in broilers.

Muscle vitamin E and liver MDA were significantly influenced by OCM, BL, and their interaction (*p* < 0.05) (Figure 2; Table 5). Vitamin E was increased by feeding BL or both OCM (4%) and BL (*p* < 0.05) (Figure 2A). Liver MDA was decreased by feeding BL or both OCM at 2% or 4% and BL (*p* < 0.05) (Figure 2B). The highest MDA level was found in broilers fed the control or OCM at 2% (*p* < 0.05) (Figure 2B).

## 4. Discussion

Most of the recent studies concluded that growth performance was not affected by including up to 10% of OCM in poultry diets [3,6,7,8,9]. This study revealed that OCM was successfully included in the diet of broilers at 4%. Moreover, by adding 4% of OCM, broilers obtained better weight gain compared to the control group. The inclusion of up to 4% of OCM in the diet did not impair broiler feed efficiency (FI and FCR). As the above-mentioned level of OCM inclusion would not yet be sufficient for today’s scenarios, the intensification of using a blend of the plant by-products has made it necessary to formulate the most cost-effective balanced feed with sound nutrition. This agrees with numerous studies that tested OCM in the diets of broilers [3,6,7,9]. The results showed enhanced growth parameters when broilers were fed OCM at 4%. However, chicks fed a diet with a high level of OCM (4%) with BL showed better growth performance and feed utilization. In the case of a high inclusion level (4%) with BL, the body weight was significantly higher than the other groups, which may result in a reduced feed conversion ratio (FCR). High digestive enzyme activity and feed palatability are also other factors that could increase feed efficiency and, accordingly, the growth of broilers [29]. Zhao et al. [11] reported that low feed intake and a low feed efficiency ratio (increased FCR) resulted from BL supplementation in the broilers’ diet. In parallel, a significant role of this probiotic bacterium on the growth, intestinal microbiota, and immunological responses in chicks has been demonstrated [14,18]. Probiotics may increase the growth and feed efficiency by increasing the secretion of amylase, protease, and lipase, which can increase the digestibility of nutrients in the animal’s gut [21,22,23,24]. The improvement of growth performance by feeding OCM with BL appears to result from an increase in the feed efficiency of broiler chickens and metabolizable energy (ME) from the diet. The reason for this increase in ME could be due to the digestion of either raw starches or soluble and insoluble non-starch polysaccharide content in OCM, as this probiotic possesses the ability to digest raw starches and to produce cellulase and xylanase, which are required for the digestion of insoluble non-starch polysaccharides [30,31]. In addition, probiotics could improve the nutritional quality of soybean meal because the trypsin inhibitor contained in unprocessed soybean is degraded by BL [32].

The abdominal fat was lowered by including OCM and/or BL in the current study. Similarly, Al-Harthi [3] stated that abdominal fat was decreased by using OCM and/or yeast as probiotics in the broilers’ diet. In this study, broilers’ feed may elevate the body fat bulk, which may be the reason for the increasing abdominal fat level in broilers fed the control diet in comparison to OCM and/or BL. Probiotics are well known for their function in facilitating the gut absorption of essential nutrients to improve the growth and, accordingly, the general health status, which means reducing the accumulation of nutrients in the gut such as abdominal fat [33].

Blood biochemical indicators relate to some enzymes activity and protein levels in the blood [34] which can reflect the physiological and immunological status of the organism [35]. GOT levels in serum can reflect liver function. When the liver is damaged, the activity will be higher than the normal range [36]. The result of this study revealed that GOT was not influenced by test diets, which indicates the safe function of OCM and/or BL in the broilers’ diet. Albumin is the highest protein in serum, which refers to increased antibody and immunity responses [37]. Compared to the control group, after the addition of OCM and/or BL, the changes of biochemical indices and blood routine indices were within the normal range, indicating that OCM and/or BL had no adverse effects on the liver, kidney, and other organs and muscles, as well as on the protein metabolism of broilers. This also proves that the addition of OCM and/or BL had no adverse effect on animal health. Furthermore, dietary OCM and/or BL supplementation in the present investigation elevated the level of total protein and ND titer, which can be attributed to the improved immunity of broilers. Olive oil is a potent immunomodulator that can improve immunity and generate more pathogen resistance [5].

By feeding OCM and/or BL, plasma HDL cholesterol concentration was increased, while plasma total cholesterol was decreased in broilers. Unfortunately, similar investigations concerning the inclusion of OCM and/or BL in broilers’ diet are very scarce. Generally, OCM has been reported to decrease the total cholesterol and to increase HDL lipids due to its content of unsaturated and polyunsaturated fatty acids [3,38,39]. Similarly, using OCM in broilers’ feed has been shown to result in low levels of total cholesterol [3,40]. The obtained results also revealed that the effect of BL on blood cholesterol levels depends on the level of OCM inclusion. The influence of probiotics in reducing the total cholesterol can be attributed to its role in breaking down the total lipids and bile acids to avoid the re-synthesis of cholesterol [24,33]. Similar results were obtained when chicks fed diets supplemented with different probiotic strains [3,41]. These studies stated that diets containing probiotic bacteria have a negative effect on plasma total cholesterol, but a positive effect on plasma high-density lipoprotein cholesterol (HDL-C) in chickens. Saleh et al. [3,41] reported that the mechanism underlying the cholesterol-lowering effect of probiotics could be due to the inhibition of 3-hydroxyl-3-methylglutaryl-coenzyme (HMG-CoA) reductase. In addition, probiotics might affect fat deposition by influencing the activities of hormone-sensitive lipase and malate dehydrogenase enzyme in adipose tissues [42].

The obtained results also revealed improved ND titer in broilers fed OCM and/or BL, which indicates an improved immunity and, consequently, resistance against infectious diseases. Balanced diets supplemented with reasonable functional feed additives usually can keep high immunity status and resistance against infectious diseases in broilers [1,2]. Probiotics, on the other hand, are beneficial microorganisms that compete with the harmful bacteria in the animals’ gut and provides the host high resistance against infectious diseases [43]. The muscle oleic, linoleic, and linolenic acids as unsaturated fatty acids were increased by OCM and/or BL in the broilers’ diet in this study. The increased levels of muscle oleic, linoleic, and linolenic acids can be attributed to the high content of OCM from unsaturated and polyunsaturated fatty acids [38,39]. The increase in oleic, linoleic, and linolenic acids in the muscle is probably due to the intestinal activities of the probiotic. Srianta et al. [44] reported that probiotics produce linolenic acid. Furthermore, BL has the ability to produce desaturase, which changes saturated fatty acids to unsaturated fatty acids [45].

Regarding the muscle vitamin E, feeding OCM and/or BL increased the level of vitamin E in the muscle of broilers, which might be involved in reducing the lipid peroxidation process in broilers and, accordingly, in reducing the oxidation [24]. Oxidative emphasis normally happens when the creation and elimination of free radicals (ROS) are unbalanced, since the oxidative damage of cultured species is directly related to the quality of the diet [46,47]. Superoxide dismutase, glutathione peroxidase, and catalase are important scavengers of ROS, protecting the body tissues from oxidative stress damage [48]. Malondialdehyde (MDA) is a product of lipid peroxides and high levels of ROS, which can cause damage to the cell’s DNA, protein, and cytoplasm [49,50]. Interestingly, broilers fed OCM and/or BL showed reduced MDA, confirming that the known antioxidant properties of this probiotic are not lost when administered orally in broilers. Like the current study, earlier reports revealed improved antioxidant response by feeding probiotics [11,51].

## 5. Conclusions

In conclusion, feeding olive cake meal with *B. licheniformis* improved growth performance, modified plasma lipid, and fatty acid profiles, as well as enhanced the health status of broiler chickens—these factors were probably influenced through improved feed efficiency and antioxidative response. Based on the results obtained, the use of BL to improve the nutritional value of OCM and to increase the feed utilization of OCM by broilers is recommended.

## Figures and Tables

**Table 1:** Composition of the experimental diets *.

Ingredients (g/kg)	Control			OCM (2 %)			OCM (4 %)		
	Starter	Grower	Finisher	Starter	Grower	Finisher	Starter	Grower	Finisher
Yellow corn	532	583	641	512	563	621	492	543	601
Soybean meal (44%)	361	305	230	361	305	230	361	305	230
Gluten (62%)	45	46	59	45	46	59	45	46	59
Soybean oil	22	27	30	22	27	30	22	27	30
Dicalcium phosphate	16	15	15	16	15	15	16	15	15
DL-Methionine	2	1.8	1.2	2	1.8	1.2	2	1.8	1.2
L-Lysine	1.3	1.4	2.4	1.3	1.4	2.4	1.3	1.4	2.4
Threonine	0.5	0.3	0.1	0.5	0.3	0.1	0.5	0.3	0.1
CaCo_3_	12	12	11	12	12	11	12	12	11
NaCl	3.5	3.5	3.5	3.5	3.5	3.5	3.5	3.5	3.5
Premix **	3	3	3	3	3	3	3	3	3
NaHCO_3_	1.5	1.5	1.6	1.5	1.5	1.6	1.5	1.5	1.6
K_2_CO_3_	0.2	0.5	2.2	0.2	0.5	2.2	0.2	0.5	2.2
Olive cake meal ***	0	0	0	20	20	20	40	40	40
Chemical analysis									
CP (%)	23.04	21.02	19.02	23.07	21.04	19.04	23.1	21.07	19.07
ME (Kcal/kg)	2951	3042	3143	2950	3041	3142	2950	3040	3141
Ca, %	0.93	0.892	0.834	0.93	0.892	0.834	0.93	0.892	0.834
Avp. P (%)	0.428	0.401	0.389	0.428	0.402	0.39	0.428	0.402	0.39
Fiber (%)	3.949	3.659	3.271	4.183	3.893	3.505	4.417	4.127	3.739
Na (%)	0.194	0.194	0.197	0.193	0.194	0.197	0.193	0.193	0.197
Cl (%)	0.252	0.251	0.25	0.251	0.25	0.249	0.25	0.249	0.249

* The basal diet fed to the chicks was formulated to meet the NRC [24] recommendations for broiler chickens; ** Premix (Hero mix^®^ ,Hero pharm, Cairo, Egypt). Composition (per 3 kg): vitamin A 12,000,000 IU, vitamin D3 2,500,000 IU, vitamin E 10,000 mg, vitamin K3 2000 mg, vitamin B1 1000 mg, vitamin B2 5000 mg, vitamin B6 1500 mg, vitamin B12 10 mg, niacin 30,000 mg, biotin 50 mg, folic acid 1000 mg, pantothenic acid 10,000 mg, manganese 60,000 mg, zinc 50,000 mg, iron 30,000 mg, copper 4000 mg, iodine 300 mg, selenium 100 mg, and cobalt 100 mg). *** Olive cake meal (OCM) analysis (crude protein (CP; 9%), metabolizable energy (ME; 3320 Kcal/kg), Ca; 0.021%, available phosphorus (Avp.; 0.29%), ether extract; 13.7%, fiber; 11.6%).

**Table 2:** Effects of feeding *Bacillus licheniformis* (BL) or/and olive cake meal (OCM) on growth performance and organ weights in broilers.

Item	Control	OCM (2%)	OCM (4%)	BL	OCM (2%)/BL	OCM (4%)/BL	*p* Value		
							BL	OCM	BL × OCM
Initial body weight (g)	46 ± 0.57	46 ± 0.41	45.5 ± 0.64	45.5 ± 0.28	45.3 ± 0.48	45.75 ± 0.63	0.061	0.12	0.23
Body weight gain (WG, g/35 day)	2010 ± 27 ^d^	2052 ± 13 ^cd^	2090 ± 34 ^bc^	2071 ± 11 ^bcd^	2137 ± 32 ^ab^	2180 ± 15 ^a^	0.041	0.032	0.002
Feed intake (g/35 day)	3487 ± 50	3428 ± 21	3447 ± 96	3410 ± 15	3455 ± 9 ± 35	3433 ± 29	0.25	0.32	0.21
Feed conversion ratio (g feed/g WG)	1.735 ± 0.01 ^a^	1.634 ± 0.02 ^ab^	1.670 ± 0.03 ^ab^	1.647 ± 0.02 ^ab^	1.617 ± 0.03 ^b^	1.575 ± 0.01 ^b^	0.034	0.041	0.035
Mortality (%)	1.66 ± 0.02	1.66 ± 0.03	1.66 ± 0.02	1.66 ± 0.02	1.66 ± 0.03	1.66 ± 0.03	0.78	0.82	0.72
Organ weight (% body weight)									
Carcass	67.9 ± 1.6	67.3 ± 1.3	68.9 ± 1.4	68.8 ± 1	69.9 ± 161	70.7 ± 1.1	0.12	0.23	0.22
Breast muscle	22.71 ± 0.31	22.37 ± 0.89	22.78 ± 0.89	23.06 ± 0.46	22.82 ± 0.64	23.13 ± 0.88	0.34	0.25	0.31
Thigh muscle	16.36 ± 0.52	16.1 ± 0.48	16.28 ± 0.53	16.45 ± 0.46	16.37 ± 0.16	16.2 ± 0.33	0.32	0.24	0.44
Liver	2.24 ± 0.12	2.32 ± 0.12	2.38 ± 0.06	2.23 ± 0.11	2.35 ± 0.21	2.39 ± 0.14	0.52	0.61	0.34
Abdominal fat	2.08 ± 0.11 ^a^	1.61 ± 0.22 ^b^	1.61 ± 0.17 ^b^	1.89 ± 0.17 ^ab^	1.47 ± 0.12 ^b^	1.42 ± 0.06 ^b^	0.025	0.011	0.033

^a–d^ Means within the same row with different superscripts differ (*p* < 0.05). Results are presented as means ± SEM.

**Table 3:** Effects of feeding *Bacillus licheniformis* (BL) or/and olive cake meal (OCM) on plasma parameters in broilers.

Item	Control	OCM (2%)	OCM (4%)	BL	OCM (2%)/BL	OCM (4%)/BL	*p* Value		
							BL	OCM	BL × OCM
GOT (mg/dL)	346 ± 11	335 ± 9	327 ± 7	320 ± 6	335 ± 10	327 ± 4	0.23	0.51	0.43
Uric acid (mg/dL)	6.97 ± 0.34	6.47 ± 0.23	6.93 ± 0.23	6.5 ± 0.3	6.6 ± 0.33	6.47 ± 0.12	0.33	0.42	0.21
Total protein (mg/dL)	3.35 ± 0.07 ^b^	3.63 ± 0.19 ^ab^	3.65 ± 0.1 1^ab^	3.33 ± 0.11 ^b^	3.80 ± 0.09 ^a^	3.85 ± 0.15 ^a^	0.021	0.032	0.011
Albumin (mg/dL)	1.85 ± 0.06 ^b^	2.05 ± 0.19 ^ab^	2.12 ± 0.1 ^a^	2.11 ± 0.05 ^a^	2.00 ± 0.07 ^ab^	2.00 ± 0.04 ^ab^	0.001	0.021	0.002
ND titer	2.17 ± 0.48 ^b^	3.67 ± 4.19 ^a^	3.83 ± 0.31 ^a^	3.33 ± 0.42 ^a^	3.17 ± 0.17 ^a^	3.50 ± 0.22 ^a^	0.001	0.024	0.0021
Total cholesterol (mg/dL)	154 ± 3 ^a^	140 ± 2 ^ab^	139 ± 7 ^b^	140 ± 5 ^ab^	137 ± 6 ^b^	131 ± 2 ^b^	0.011	0.001	0.023
HDL-cholesterol (mg/dL)	80 ± 3 ^b^	92 ± 2 ^a^	93 ± 2 ^a^	81 ± 3 ^b^	93 ± 1 ^a^	95 ± 1 ^a^	0.025	0.034	0.029

^a–b^ Means within the same row with different superscripts differ (*p* < 0.05). Results are presented as means ± SEM. Glutamic oxalacetic transaminase (GOT), Newcastle disease (ND), and high-density lipoprotein (HDL).

**Table 4:** Two-way ANOVA (*p*-value) of the muscle fatty acids (oleic acid, arachidonic acid, linoleic acid, and linolenic acid) in broilers fed *Bacillus licheniformis* (BL) or/and olive cake meal (OCM).

	Two-Way ANOVA (*p*-Value)		
	BL	OCM	BL × OCM
Oleic acid (mg/100g fat)	0.03	0.01	0.043
Arachidonic acid (mg/100g fat)	0.13	0.24	0.12
Linoleic acid (mg/100g fat)	0.012	0.02	0.031
Linolenic acid (Mg/100g fat)	0.041	0.012	0.042

**Table 5:** Two-way ANOVA (*p*-value) of the muscle vitamin E and liver MDA in broilers fed *Bacillus licheniformis* (BL) or/and olive cake meal (OCM).

	Two-Way ANOVA (*p*-Value)		
	BL	OCM	BL × OCM
Vit E (mg/100g muscle)	0.031	0.042	0.012
MDA (nmol/g liver)	0.001	0.032	0.0012
